# Silica nanoparticles are less toxic to human lung cells when deposited at the air–liquid interface compared to conventional submerged exposure

**DOI:** 10.3762/bjnano.5.171

**Published:** 2014-09-19

**Authors:** Alicja Panas, Andreas Comouth, Harald Saathoff, Thomas Leisner, Marco Al-Rawi, Michael Simon, Gunnar Seemann, Olaf Dössel, Sonja Mülhopt, Hanns-Rudolf Paur, Susanne Fritsch-Decker, Carsten Weiss, Silvia Diabaté

**Affiliations:** 1 Institute of Toxicology and Genetics, Karlsruhe Institute of Technology, Campus North, Hermann-von-Helmholtz-Platz 1, 76344 Eggenstein-Leopoldshafen, Germany; 2 Institute for Meteorology and Climate Research, Karlsruhe Institute of Technology, Campus North, Hermann-von-Helmholtz-Platz 1, 76344 Eggenstein-Leopoldshafen, Germany; 3 Institute of Biomedical Engineering, Karlsruhe Institute of Technology, Campus South, Kaiserstraße 12, 76128 Karlsruhe, Germany; 4 Institute for Technical Chemistry, Karlsruhe Institute of Technology, Campus North, Hermann-von-Helmholtz-Platz 1, 76344 Eggenstein-Leopoldshafen, Germany

**Keywords:** aerosol, air–liquid interface, dose, silica nanoparticles, toxicity

## Abstract

**Background:** Investigations on adverse biological effects of nanoparticles (NPs) in the lung by in vitro studies are usually performed under submerged conditions where NPs are suspended in cell culture media. However, the behaviour of nanoparticles such as agglomeration and sedimentation in such complex suspensions is difficult to control and hence the deposited cellular dose often remains unknown. Moreover, the cellular responses to NPs under submerged culture conditions might differ from those observed at physiological settings at the air–liquid interface.

**Results:** In order to avoid problems because of an altered behaviour of the nanoparticles in cell culture medium and to mimic a more realistic situation relevant for inhalation, human A549 lung epithelial cells were exposed to aerosols at the air–liquid interphase (ALI) by using the ALI deposition apparatus (ALIDA). The application of an electrostatic field allowed for particle deposition efficiencies that were higher by a factor of more than 20 compared to the unmodified VITROCELL deposition system. We studied two different amorphous silica nanoparticles (particles produced by flame synthesis and particles produced in suspension by the Stöber method). Aerosols with well-defined particle sizes and concentrations were generated by using a commercial electrospray generator or an atomizer. Only the electrospray method allowed for the generation of an aerosol containing monodisperse NPs. However, the deposited mass and surface dose of the particles was too low to induce cellular responses. Therefore, we generated the aerosol with an atomizer which supplied agglomerates and thus allowed a particle deposition with a three orders of magnitude higher mass and of surface doses on lung cells that induced significant biological effects. The deposited dose was estimated and independently validated by measurements using either transmission electron microscopy or, in case of labelled NPs, by fluorescence analyses. Surprisingly, cells exposed at the ALI were less sensitive to silica NPs as evidenced by reduced cytotoxicity and inflammatory responses.

**Conclusion:** Amorphous silica NPs induced qualitatively similar cellular responses under submerged conditions and at the ALI. However, submerged exposure to NPs triggers stronger effects at much lower cellular doses. Hence, more studies are warranted to decipher whether cells at the ALI are in general less vulnerable to NPs or specific NPs show different activities dependent on the exposure method.

## Introduction

Amorphous SiO_2_ nanoparticles (NPs) are regarded as only little pathogenic. However, it has been shown that the inhalation of silica NPs induces transient inflammation in rats [1–2]. Meanwhile there are numerous reports which also demonstrate adverse effects of amorphous silica NPs in vitro, e.g., in macrophages [3–5], in lung epithelial cells [5–8] and in co-cultures of both cell types [9]. However, in vitro exposure of lung cells under submerged conditions does not reflect the physiological situation in the lung where cells are directly exposed to an aerosol. Especially our findings of a strong inhibitory effect of serum proteins on NP toxicity show how the standard cell culture model generates artefacts and might lead to wrong conclusions [5]. Additionally, particle collection and resuspension in medium may change their physico-chemical properties and the particle dose delivered to the cells under submerged conditions is often unclear due to differences in agglomeration and sedimentation of suspended NPs. In vitro experiments at the air–liquid interface (ALI) are therefore of utmost relevance.

Although exposure of cells at the air–liquid interface (ALI) represents a more realistic exposure scenario compared to submerged exposure only few research papers are found in the literature, in particular for silica NPs [10]. This is due to the need for more sophisticated laboratory equipment, technical know-how and, additionally, for the generation of a nanomaterial aerosol in a reproducible manner. A recent review prepared by toxicologists and aerosol scientists states the urgent need for further developments of in vitro cell exposure studies including those at the air–liquid interface [11]. Advantages of ALI exposures are (a) the modification of particles by filter collection and resuspension in medium are avoided, (b) the nanoparticle dose interacting with the cells can be controlled more precisely and (c) the exposure of lung cells at ALI resembles an in vivo inhalation more closely. Several exposure systems for the controlled exposure of cells to particles at the ALI have recently been developed. Most ALI systems described in the literature rely on the deposition of nanoparticles by diffusion mechanisms [12–17].

Due to the small size of the nanoparticles the mass and surface doses that can be applied on the cell surface through ALI exposures are very low compared to the typical “lowest observed adverse effect levels” (LOAEL) derived from submerged experiments [18]. One approach to increase the applied dose is the use of nanoparticle agglomerates. However, especially in the size regime between 100 nm and 500 nm deposition efficiencies of ALI exposure chambers based on diffusion or gravitational settling are usually very low [19]. One approach to increase the deposition rates is the use of an electrostatic field [20–23]. This enables deposition efficiencies of up to 100% for charged particles [11]. de Bruijne et al. [21] used a corona charger for efficient charging of aerosol particles and did not observe adverse effects on A549 cells by the trace gases formed in the corona. However, for particle sizes below 50 nm, the probability to be charged becomes low [24] and hence the deposition efficiency of such systems decreases.

In this study we used a well-characterised commercially available exposure chamber system (VITROCELL^®^ SYSTEMS) that we equipped with electrodes to enhance deposition by applying an electrostatic field (referred to in the following as ALI deposition apparatus, ALIDA) [18]. Aerosols with well-defined particle sizes and concentrations were generated by using commercial electrospray generators or atomizers. The deposited dose was determined by using transmission electron microscopy (TEM) and in case of labelled NPs by fluorescence analyses. Industrial SiO_2_ NPs (Aerosil^®^200, Evonik) produced by flame synthesis and SiO_2_ NPs produced by the Stöber method (Postnova Analytics, Landsberg) were used to test the biological responses in A549 cells with and without an electrostatic field at the ALI and under submerged conditions.

## Results and Discussion

### Aerosols

**Figure 1:**
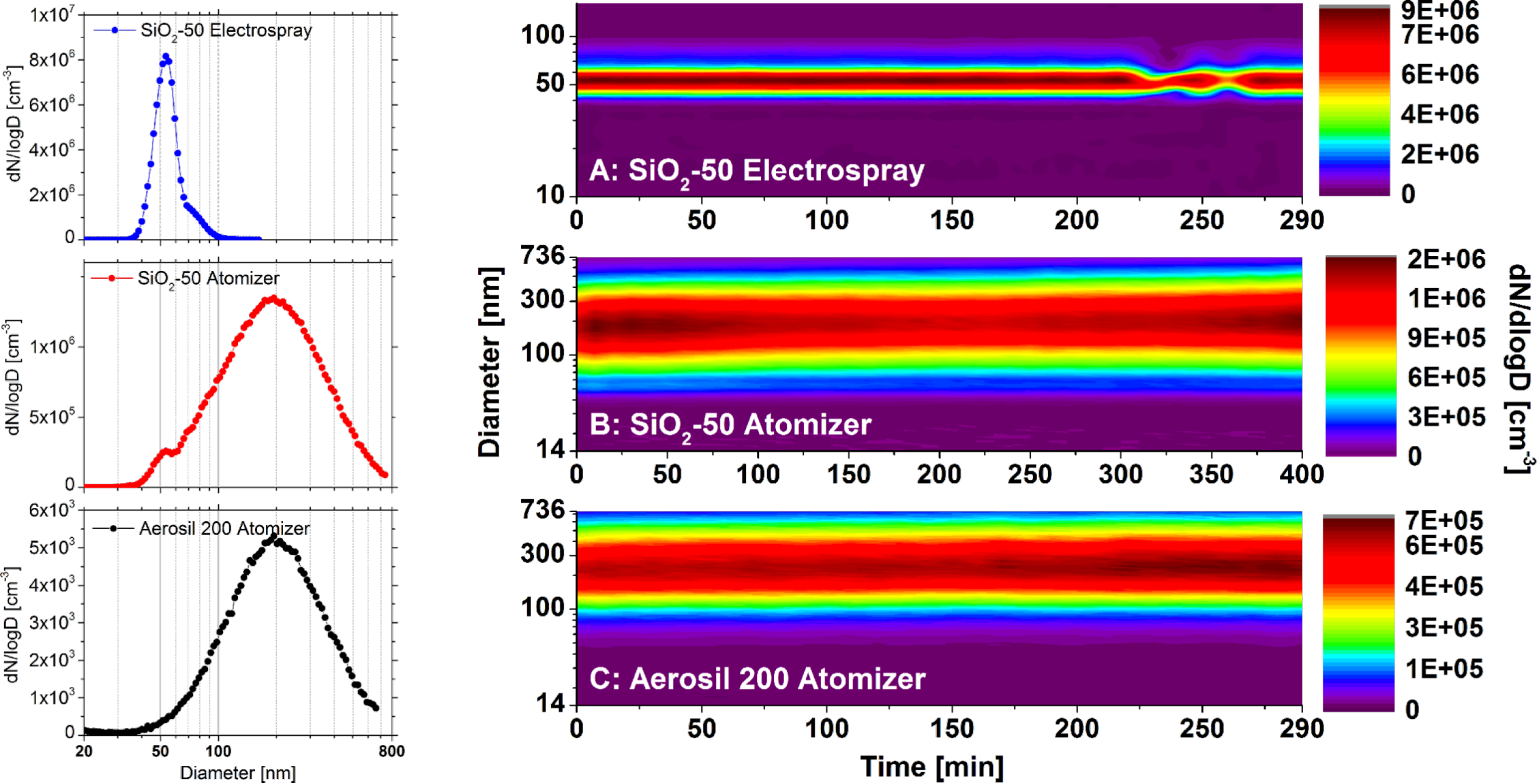
Temporal evolution of SiO_2_ particle size distributions generated for cell exposure. (A) From an aqueous solution of SiO_2_-50 nm monomers (Stöber synthesis, Postnova) high number concentrations of airborne SiO_2_-50 nm monomers with a narrow size distribution were generated by electrospray. The atomizer, however, generated from the same nanoparticle suspension large agglomerates (B) allowing for the deposition of high mass doses. Large agglomerates with a broad size distribution were also generated by atomizing Aerosil200 (C). Initial size distributions are given on the left hand side of each time chart.

The aerosols were generated by two different methods: an atomizer and electrospray. By using the electrospray method it was possible to generate an aerosol containing monomers and small agglomerates of SiO_2_-50 nm NPs with a high number concentration and a narrow size distribution (Figure 1A). This method was however not applicable for the dispersion of Aerosil200 suspensions which contain aggregates that were too large for a stable operation of the electrospray in the cone jet mode. Therefore, an atomizer was used that delivered a relatively large and broad droplet size distribution [25] and, with Aerosil200, an aerosol with constant but broad size distribution (Figure 1C). Furthermore, dispersion of SiO_2_-50 nm NPs with the atomizer allowed for the generation of large agglomerates (Figure 1B) which resulted in high mass and surface doses.

### Particle deposition

**Figure 2:**
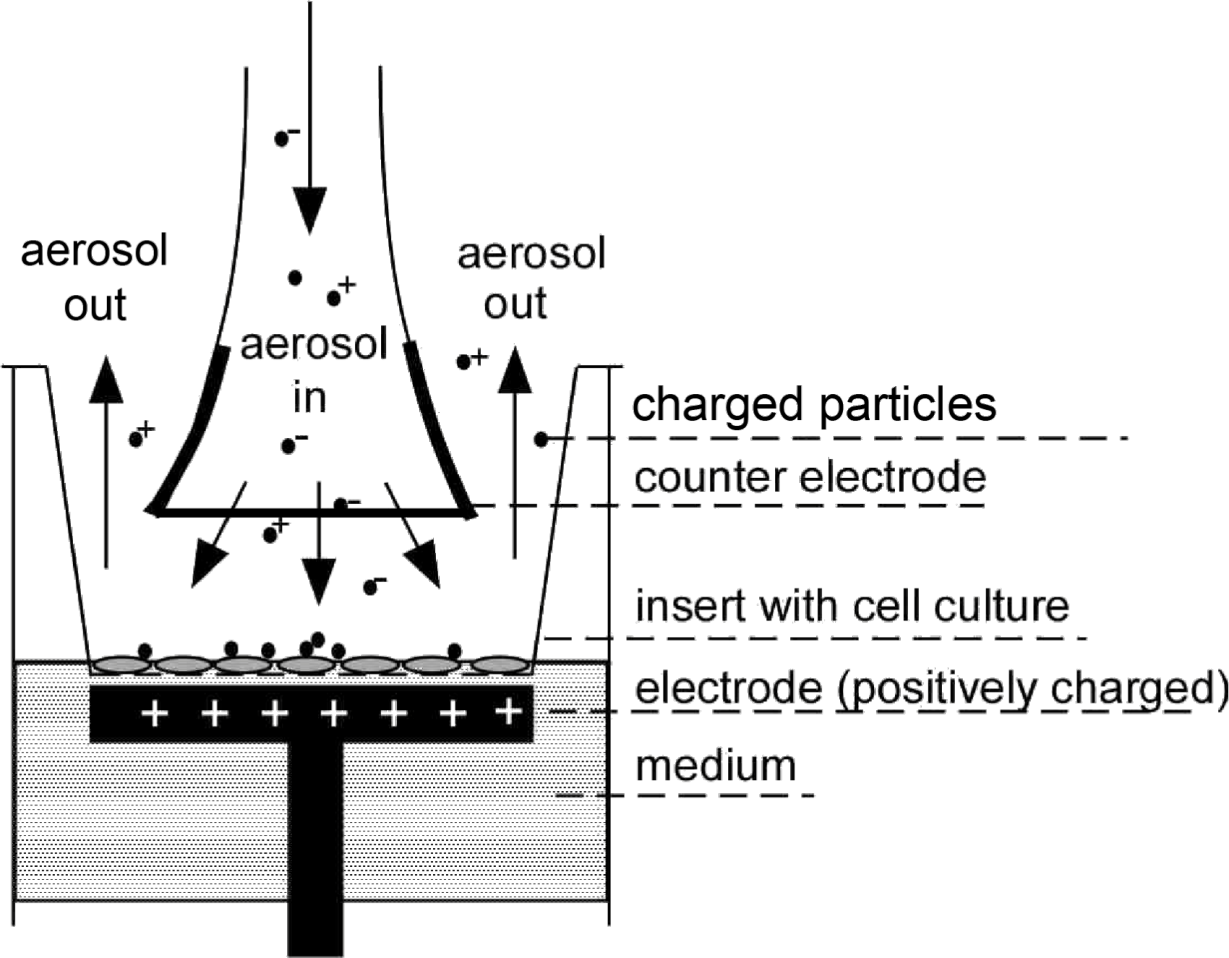
Schematic view of the Vitrocell^®^ exposure chamber modified with an electrode for electrostatic particle deposition.

The aerosol was directed into the ALI deposition apparatus (ALIDA) as described in section Experimental and the particles were deposited on Transwell membranes covered with test cells without or with applying an electrostatic field (Figure 2).

In order to increase the deposition efficiency the exposure chambers were modified with an electrode similar to the setup presented by Savi et al. [20]. In contrast to Savi et al. who used an alternating electrical field, during the exposure experiments a constant voltage of 1 kV was used in this study. Furthermore, no insulation between electrode and cell medium was installed, so that the cells themselves can be considered as an equipotential surface and electrode, respectively. This makes a close distance between electrode and cells unnecessary and allows for the use of larger medium volumes. As counter electrode a fine conducting mesh was placed 2 mm above the cell surface at the end of the aerosol inlet.

**Figure 3:**
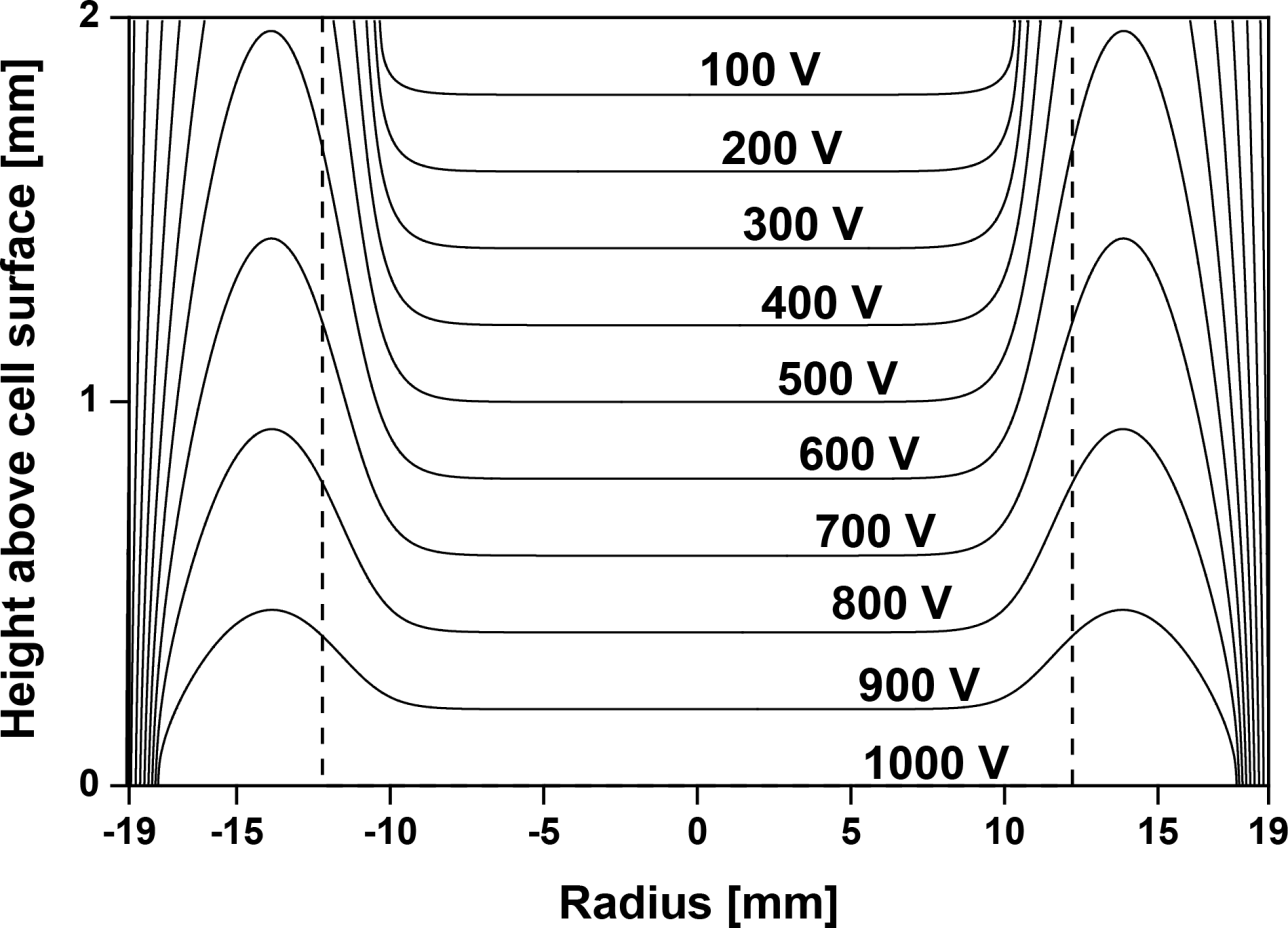
Electrostatic potential within the exposure chambers. Assuming a flat equipotential surface the electrostatic potential was 1 kV at the cell level. The dashed lines indicate the borders of the Transwell membrane. The field profile was simulated with SimIon (SIS, v8). Isolating parts of the Transwell insert and chamber were neglected.

The electrical field was almost homogeneous with an axial strength of 500 V·mm^−1^. A computer simulation revealed that only at the outer border of the Transwell membranes (more than 10 mm distance from the center of the membrane) a significant inhomogeneity of the field is expected due to increasing radial components in the electrical field strength (Figure 3). These radial components however, cause a force in radial direction and hence provide a more homogeneous particle deposition on the cell surface than a purely homogeneous field profile.

For determination of the cellular dose cell-free Transwell membranes carrying one or more grids for transmission electron microscopy (TEM) were exposed to the aerosol under the same conditions as the cells and analysed for particle loading.

### Deposition of SiO_2_-50 nm NPs

**Figure 4:**
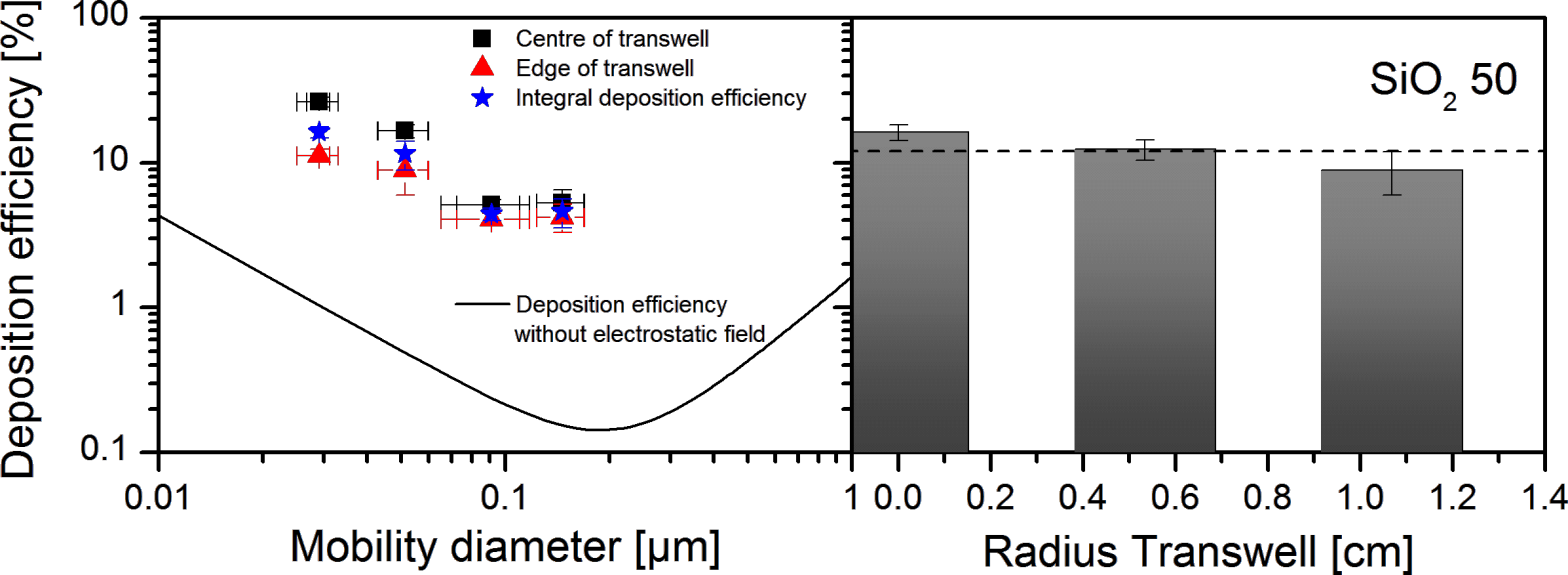
Deposition efficiency of SiO_2_ monomers depending on particle size (left plot) with electrostatic field (symbols) and without electrostatic field (SiO_2_ particle characteristics and drawn line as in Comouth et al. [18] and deposition efficiency of SiO_2_-50 nm monomers in dependence of the position on the Transwell surface (plot on the right)). SiO_2_-50 nm monomers were dispersed by electrospray and deposited on a Transwell surface with electrostatic field. The deposited mass was calculated from particle loadings on TEM grids at three different radial positions on the Transwell surface from the center to the edge. The dashed line indicates the averaged deposition efficiency. The results indicate a moderate but significant linear decrease of the particle loading between the center and the edge of the Transwell membranes.

The SiO_2_-50 nm particles were produced by the Stöber method and provided by the manufacturer as an aqueous suspension containing monomeric particles. For preparing an aerosol the suspension was diluted with water by factors of 3.6 and 7.7 for electrospray and the atomizer, respectively. The electrospray generated aerosol particles with a mean mobility size of (53 ± 1) nm (σ = 1.2) and a typical number concentration of (1.3 ± 0.2) × 10^6^ cm^−3^. The uncertainties stated are, if not defined otherwise, standard deviations of all experiments done and the parameter σ characterises the standard deviation of a log-normal size distribution. After the exposure of empty Transwell membranes loaded with TEM grids to SiO_2_-50 nm monomers generated by electrospray and deposited with the electrostatic field a total number dose of (6 ± 2) × 10^8^ cm^−2^ was determined by analysis of the TEM pictures (Table 1). Furthermore, a mean projection equivalent diameter of the particles of (54 ± 3) nm (σ = 1.1) was measured on the TEM grids in very good agreement with the diameter of the airborne particles. Since the particles were not homogeneously distributed over the entire Transwell membrane (Figure 4) the total number dose corresponds to a mean particle number dose. Compared to the total applied particle number this dose is equivalent to a deposition efficiency of (11 ± 3)% which is 22-fold higher compared to 0.5% achieved with this exposure chamber without electrostatic field for particles of this size [18]. However, due to the small monomer size the corresponding mass and surface dose after 5 h of exposure only amounts to (0.14 ± 0.05) µg·cm^−2^ and (0.08 ± 0.03) cm^2^·cm^−2^, respectively (see Table 1).

**Table 1:** Characteristics of the particles, the aerosol generation and deposition with electrostatic field.

	Aerosil200 atomizer	SiO_2_-50 nm atomizer	SiO_2_-50 nm electrospray

primary particle size (TEM)	(7–100) nm^a^	(54 ± 3) nm^b^	(54 ± 3) nm^b^
specific surface area	200 m^2^·g^−1 c^	60 m^2^·g^−1 d^	60 m^2^·g^−1 d^
**aerosol generation**			
concentration of the suspension used for aerosol generation	1 mg·mL^−1^	3.25 mg·mL^−1^	7 mg·mL^−1^
mean (agglomerate) mobility diameter in air (SMPS)	(279 ± 10) nm	(230 ± 10) nm	(53 ± 1)nm
agglomerate fraction^e^	100%	92%	7%
**particle deposition**			
exposure duration	5 h	7 h	5 h
mass dose (TEM)	(52 ± 26) µg·cm^−2^	(117 ± 46) µg·cm^−2^	(0.14 ± 0.05) µg·cm^−2^
surface dose (TEM)	(104 ± 52) cm^2^·cm^−2^	(70 ± 28) cm^2^·cm^−2^	(0.08 ± 0.03) cm^2^·cm^−2^
number dose (TEM)	(2.0 ± 0.8) × 10^8^ cm^−2^	(1.4 ± 0.3) × 10^9^ cm^−2^	(6 ± 2) × 10^8^ cm^−2^
deposition efficiency	—	—	(11 ± 3)%
experiments per endpoint	3	2	2

^a^The manufacturer calculated a primary particle size of 12 nm from the BET surface. ^b^The manufacturer states a size of 70 nm analysed by DLS. ^c^Given by the manufacturer. ^d^Value was estimated from the primary particle size. ^e^Obtained by analysis of size distributions and TEM pictures [18].

**Figure 5:**
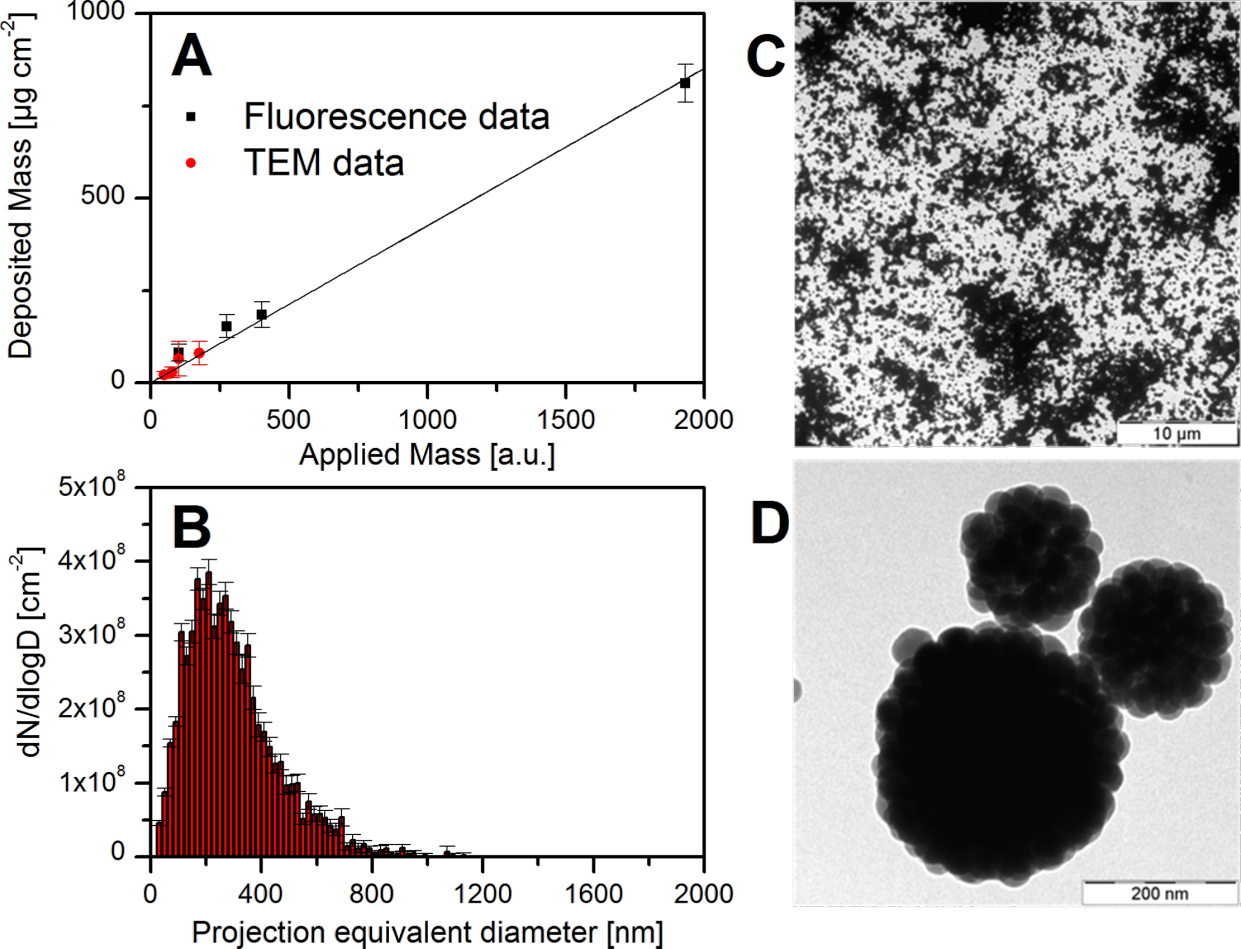
Characteristics of deposited SiO_2_-50 nm agglomerates. Aerosols of fluorescently labeled SiO_2_-50 nm agglomerates were generated by an atomizer and deposited on Transwell membranes with an applied electrostatic field. The deposited mass was either detected by measuring fluorescence intensity or using TEM analysis of particle loaded grids. (A) shows the deposited mass as a function of the applied mass by the two detection methods. The size distribution of the corresponding particles on a TEM grid is shown in (B). (C) shows the particle loading on TEM grids after seven hours of exposure corresponding to a mass dose of (117 ± 46) µg·cm^−2^ and (D) representative clusters at higher magnification indicating the spherical agglomerate shape. Please note that the applied mass in (A) is given in arbitrary units (third power of the mobility equivalent diameter) and neither the effective density nor the total particle size range was considered. Hence the slope of the linear fit is not equal to the mean mass deposition efficiency.

In contrast to the electrospray the atomizer delivered an agglomerate fraction of about 92% (data not shown) with a mean mobility diameter of (230 ± 10) nm (σ = 1.8) (Figure 1) and typical number concentrations of (7 ± 1) × 10^5^ cm^−3^. Compared to the particles in the aerosol a slightly larger mean projection equivalent diameter of (270 ± 30) nm (σ = 1.9) was observed to be deposited (Figure 5B) with a total number dose of (1.4 ± 0.3) × 10^9^ cm^−2^. Reasons for these different sizes are the size-dependent deposition efficiency and different equivalent diameters that were used for size classification with SMPS and TEM, respectively. The latter one, however, may be negligible due to the almost compact spherical structure of the agglomerates (Figure 5D). Small agglomerates showed structures similar to the clusters described by Cho et al. [26] and Manoharan et al. [27]. More than 95% of the mass however were provided by agglomerates larger than 200 nm mobility equivalent diameter. Agglomerates of this size contain more than 40 monomers so that errors due to different effective densities of small agglomerates and monomers are also assumed to be negligible. The mass dose resulting from analysis of the TEM micrographs as a function of the applied mass is shown in Figure 5A. Within the uncertainties TEM and fluorescence data are in very good agreement and can be described by a linear function of the applied particle mass. For the cell exposures a mean mass dose of (117 ± 46) µg·cm^−2^ follows from this function. This is within the dose range of classical nanotoxicology studies under submerged conditions and beyond the lowest observed adverse effect level (LOAEL) defined for, e.g., Aerosil200 in lung epithelial cells [5].

### Deposition of Aerosil200

**Figure 6:**
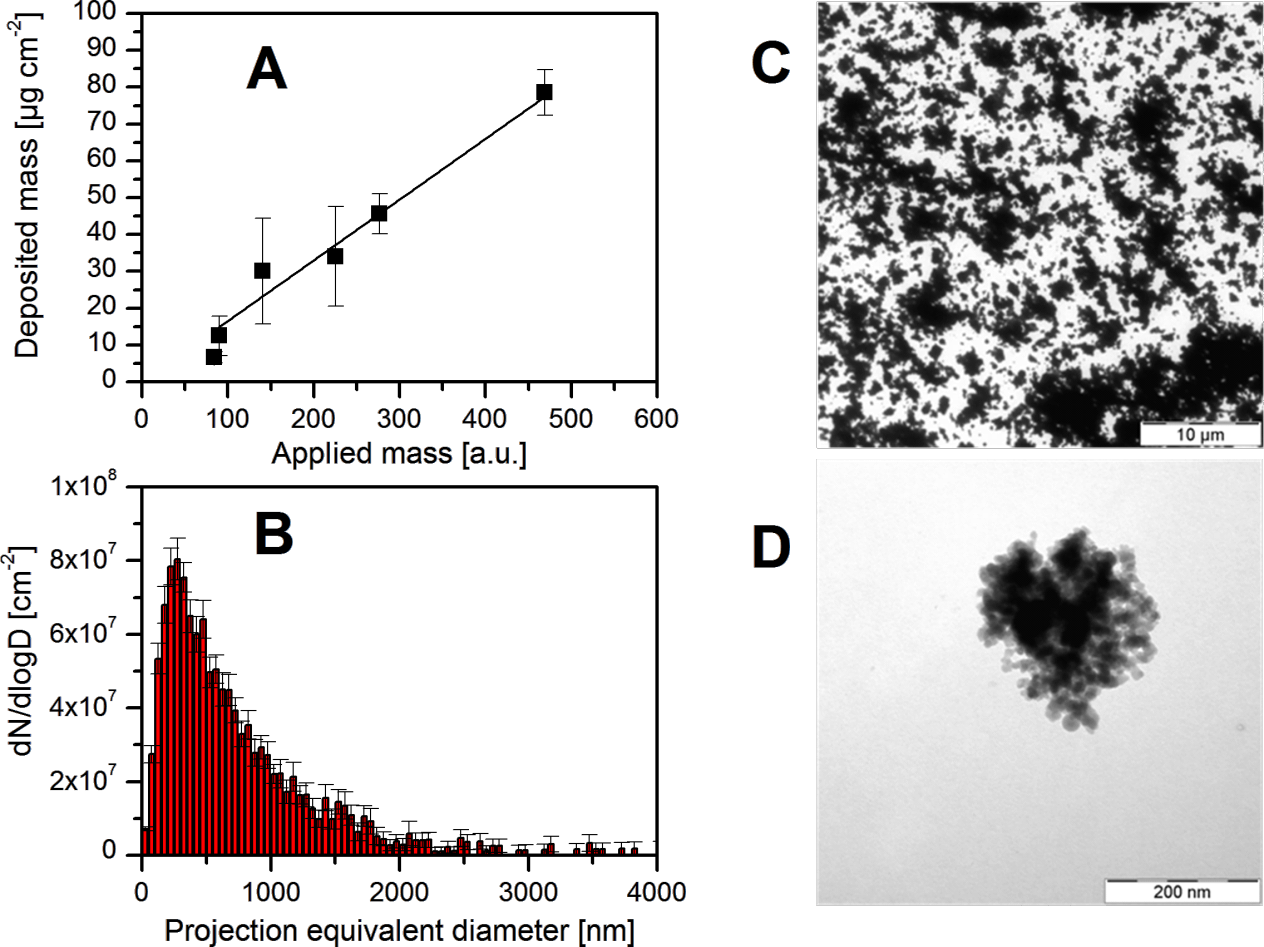
Deposited mass (derived from the number of particles) of Aerosil200 agglomerates as a function of the applied mass (A), size distribution of the corresponding particles (B), as well as the particle loading on a TEM grid after five hours of exposure corresponding to a mass dose of (52 ± 26) µg·cm^−2^ (C), and a representative cluster indicating the agglomerate shape (D). Please note that the applied mass in (A) is given in arbitrary units (third power of the mobility equivalent diameter) and neither the effective density nor the total particle size range was considered. Hence the slope of the linear fit is not equal to the mean mass deposition efficiency.

Aerosil200 are industrial SiO_2_ NPs produced by flame synthesis and provided as a powder. The manufacturer states a mean primary particle size of 12 nm. However, our analyses by TEM revealed monomeric particle sizes between 7 and 100 nm almost exclusively forming larger agglomerates. For preparing an aerosol the powder was suspended in water and ultrasonicated. The hydrodynamic diameter determined by DLS in water was (215 ± 25) nm as also reported previously [5]. The mean sizes of the aerosolized and deposited Aerosil200 agglomerates were (279 ± 10) nm (σ = 1.8) mean mobility equivalent and (491 ± 40) nm (σ = 2.25) mean projected area equivalent diameter, respectively (Figure 6B), and hence even larger compared to the SiO_2_-50 nm particles (Table 1). Please note that the larger difference between the two given equivalent particle diameters is caused by the fluffy structure of the Aerosil200 agglomerates since the measured mobility equivalent diameter corresponds to the diameter of a hypothetical compact sphere with the same dynamic mobility as the agglomerates and the projected area equivalent diameter to that of a circle with the same area as the projected area of the agglomerates under the microscope [28]. Due to smaller deposited number concentrations of (2.0 ± 0.8) × 10^8^ cm^−2^, the corresponding mean mass dose determined from TEM micrographs and measured effective densities was only (52 ± 26) µg·cm^−2^. The analysis of TEM pictures assuming compact agglomerates results in the upper limit of this mass dose (78 µg·cm^−2^). However, the true deposited dose is presumably lower as the effective densities of larger agglomerates are decreased. Aerosol particle mass (APM) measurements delivered effective densities of (0.81 ± 0.02), (0.53 ± 0.02) and (0.39 ± 0.01) g·cm^−3^ for particle mobility equivalent sizes of 80 nm, 250 nm, and 800 nm, respectively, which is 34–68% less than estimated when considering the rather compact SiO_2_-50 nm agglomerates. This is in agreement with TEM micrographs that show a more complex cluster structure compared to SiO_2_-50 nm agglomerates that cannot be described by a packing of hard spheres (Figure 6D). We assume this structure is caused by strongly bonded agglomerates and aggregates present in Aerosil200 after its synthesis in gas phase as reported by Seipenbusch et al. [29]. Such strong particle interactions prevent the monomers from restructuring within the atomized droplets. Hence Aerosil200 agglomerates are rather composed of irregular shaped aggregates [30] than of spherical monomers behaving like hard spheres. For this reason the actual effective density decreases with increasing agglomerate size (Figure S1, Supporting Information File 1). Therefore, the minimal dose of 26 µg·cm^−2^ was calculated considering only the lowest effective density for the largest agglomerates, which, however, is obviously underestimating the final deposited mass. Hence, the actual dose will be within the calculated extremes and is given as (52 ± 26) µg·cm^−2^.

### Biological effects

For the determination of biological effects the Transwell inserts covered with a confluent layer of A549 cells were exposed to filtered and unfiltered aerosol. Exposure to the unfiltered aerosol of Aerosil200 NP and SiO_2_-50 nm NP agglomerates led to deposited doses of 52 µg·cm^−2^ and of 117 µg·cm^−2^, respectively (see Table 1), which roughly leads to similar surface doses of about 104 cm^2^·cm^−2^ for Aerosil200 and 70 cm^2^·cm^−2^ for SiO_2_-50 nm NPs.

For the comparison with submerged exposure to silica NPs, the cells in Transwell inserts were treated with 50 µg·mL^−1^ NP suspensions in medium without FCS. The cellular dose was estimated according to the computational model of Hinderliter et al. [31]. The particle dose delivered to the cells is determined by diffusion and sedimentation processes, which are dependent on the particle sizes [31]. Aerosil200 NPs are detected as agglomerates in cell culture medium with an average hydrodynamic diameter of (220 ± 6) nm in the freshly prepared suspension and remain stable up to 24 h (Table S1, Supporting Information File 1). In contrast, SiO_2_-50 nm NPs are mainly present as (72 ± 19) nm NPs directly after dispersion but are also detected to a minor extent as larger agglomerates of about 2 μm after 24 h (Table S1, Supporting Information File 1). The final calculated deposited mass after 24 h is nearly identical for both Aerosil200 and SiO_2_-50 nm NP suspensions and amounts to 7.0 µg·cm^−2^ (Figure S2, Supporting Information File 1).

**Figure 7:**
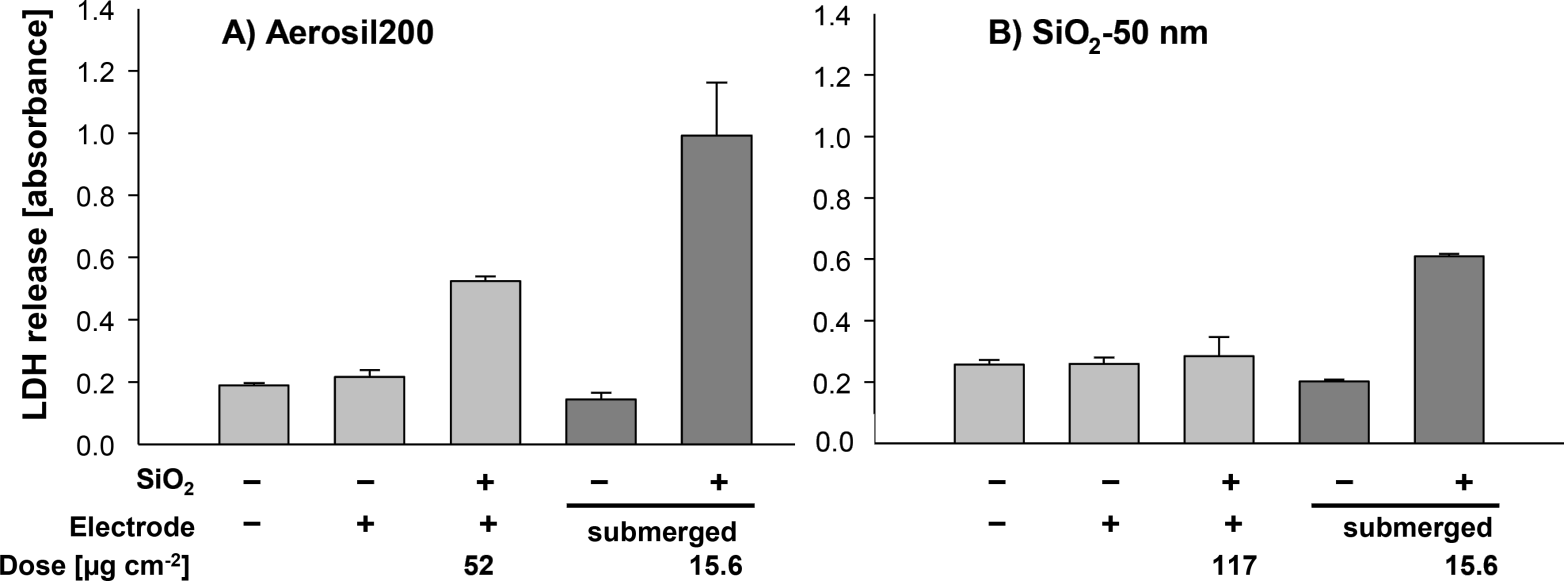
Effects of ALI and submerged exposure to (Aerosil200) and SiO_2_-50 nm agglomerate NPs (B) on the integrity of the membrane of A549 cells determined by LDH release. A549 cells were exposed to filtered air or to aerosols containing Aerosil200 NPs (A) or SiO_2_-50 nm NPs (B) under ALI conditions with application of an electrode (+). Controls were exposed to filtered air with or without electrode (−). The mass doses after ALI exposure were 52 µg·cm^−2^ for Aerosil200 NPs after 5 h and 117 µg·cm^−2^ for SiO_2_-50 nm NPs after 7 h. Subsequently, cells were post-incubated in medium without FCS and supernatants were processed 24 h after the onset of exposure. For comparison, cells were treated for 24 h with an estimated maximal dose of 15.6 µg·cm^−2^ (50 µg·mL^−1^) applied as suspension in medium without FCS or with medium alone. Results are means ± s.e.m. of 4 to 6 samples originating from three (A) and two independent experiments (B).

Figure 7 shows that the electrical field and the exposure to filtered air had no effect on the release of lactate dehydrogenase (LDH) into the medium. Enhanced levels of LDH indicate membrane damage, which leads to cell death. LDH values of the ALI-exposed control samples were similar to those detected under submerged conditions. In contrast, exposure to silica NPs induced strong LDH release under submerged exposure conditions. The LDH release after submerged exposure to 15.6 µg·cm^−2^ (50 µg·mL^−1^) Aerosil200 indicates 100% cell lysis as it was comparable to the LDH release from Triton X-100-treated cells, which were used as positive controls in some experiments (data not shown). However, cells exposed to NPs in the ALI system were less sensitive compared to submerged exposure. Under both conditions, SiO_2_-50 nm NPs were less effective in reducing the membrane integrity than Aerosil200 NPs.

**Figure 8:**
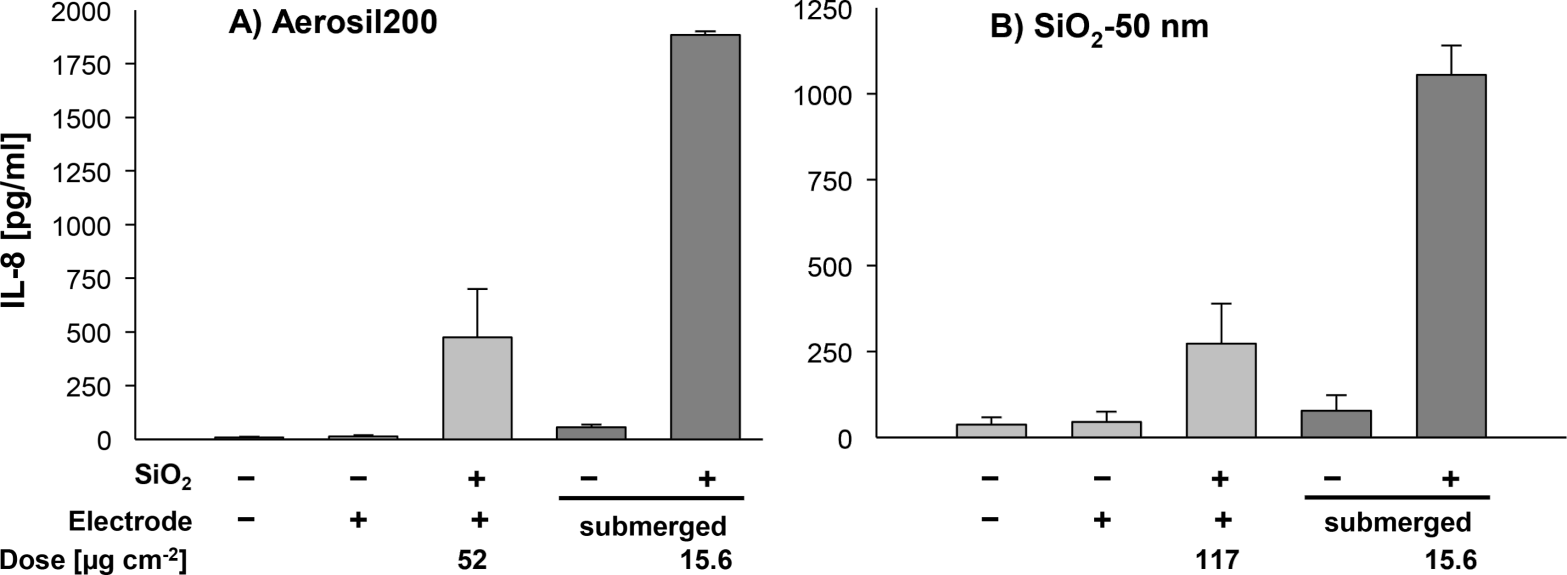
Release of IL-8 from A549 cells after submerged and ALI exposure to Aerosil200 and SiO_2_-50 nm NP agglomerates. A549 cells were exposed to filtered air or to an aerosol containing Aerosil200 NPs (A) and SiO_2_-50 nm NPs (B) under ALI or submerged conditions as described in Figure 7 above. IL-8 concentrations in the medium are represented as means ± s.e.m. of 4 to 6 samples originating from three (A) and two independent experiments (B).

The medium was also analysed for the release of the pro-inflammatory cytokines IL-8 and IL-6. Figure 8 shows, that the electrical field and the exposure to clean air had no effect on IL-8 release. The results at the ALI were qualitatively comparable with those obtained under submerged conditions. Exposure to silica NPs induced a strong release of IL-8 under submerged exposure conditions. Cells exposed to NPs at the ALI, however, released much lower IL-8 levels. Again under both conditions, as also seen for the release of LDH above, SiO_2_-50 nm NPs were less effective than Aerosil200 NPs. IL-6 release was not detected after ALI exposure to silica NPs and was only moderately enhanced after submerged exposure (data not shown). For comparison, a positive control for IL-6 and IL-8 release has been previously analysed under submerged conditions in medium without FCS [5]. Lipopolysaccharide (LPS, 10 µg·mL^−1^) induced the release of about 1000 pg·mL^−1^ of IL-8 and 10 pg·mL^−1^ of IL-6 after 24 h, respectively.

**Figure 9:**
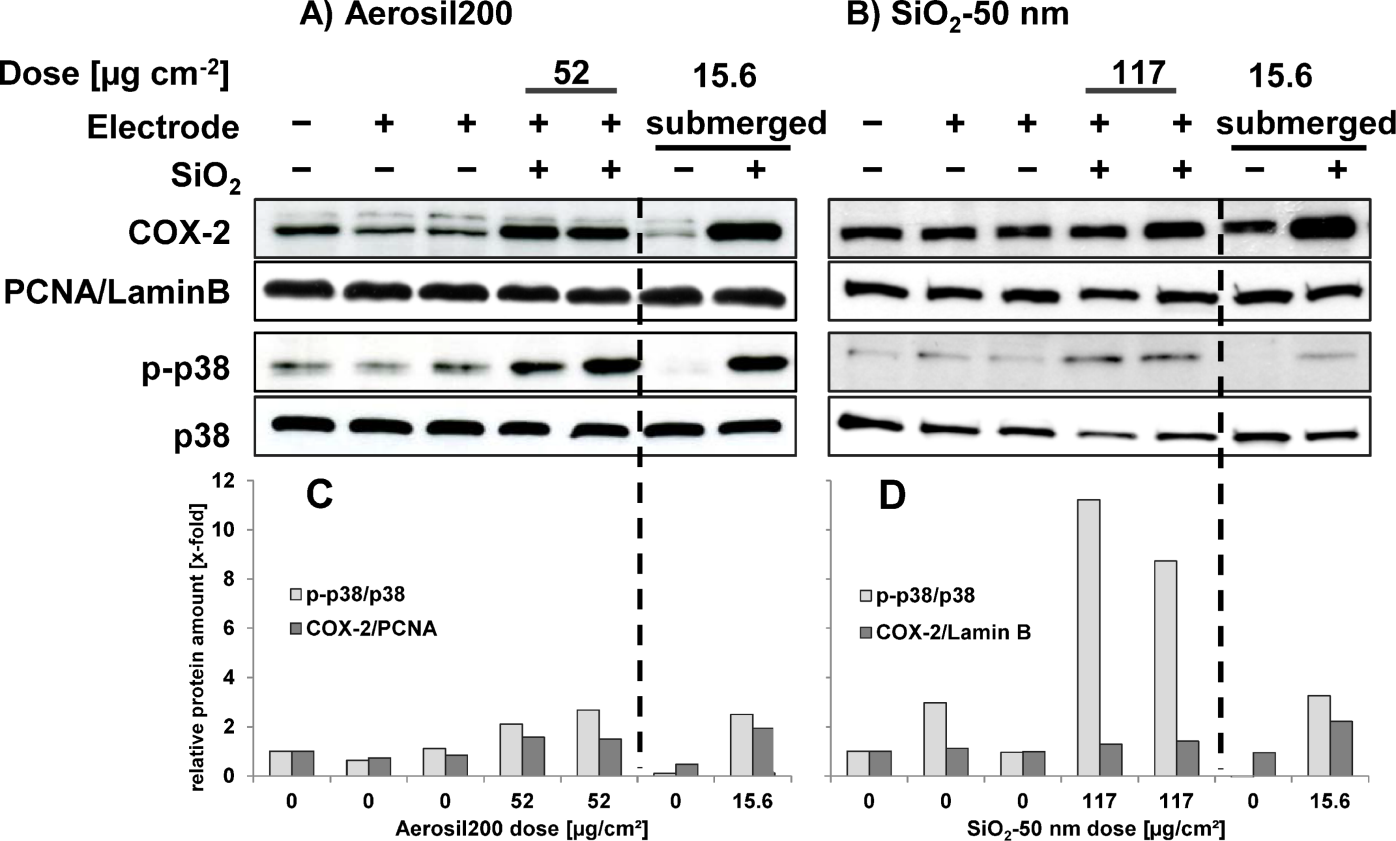
Induction of COX-2 und phosphorylation of p38 in A549 cells after SiO_2_-NP treatment under submerged and ALI conditions. A549 cells were exposed to filtered air or to an aerosol containing Aerosil200 NPs (A) and SiO_2_-50 nm NPs (B) as described in Figure 7 above. COX-2 and phospho-p38 were detected in whole cell lysates by Western blots. PCNA (left panel), Lamin B (right panel) and total p38 were used as loading controls, as indicated. The results shown are representative for three (A) and two independent experiments (B). (C) and (D) show the quantification of the p-p38 and COX-2 bands normalized to the loading controls (total p38 and PCNA or Lamin B, respectively), which are compared to the ALI controls exposed to clean air without electrode (which are set to 1).

To analyse the biological effects of NPs at earlier points in time, cells were lysed immediately after ALI or submerged exposure. Phosphorylation of the MAP kinase p38 and induction of the inflammatory protein COX-2 were determined by Western blot. Figure 9A and Figure 9C show that Aerosil200 NPs induced similar levels of p38 phosphorylation and COX-2 after submerged and ALI exposure although the deposited dose under submerged conditions was lower than at the ALI. Similar to the findings described above for the release of LDH and IL-8, induction of COX-2 by SiO_2_-50 nm NPs was also less pronounced when compared to Aerosil200 NPs (Figure 9B,D). However, the relative phosphorylation of p38 seems to be enhanced at the ALI when compared to Aerosil200 or to submerged exposure. As p38 phosphorylation was detected after 7 h of exposure whereas LDH and IL-8 release were monitored after 24 h, a more detailed kinetic analysis is needed to substantiate a possibly different regulation of this biomarker in response to SiO_2_-50 nm NPs.

Our studies on the biological effects of Aerosil200 under submerged conditions confirm results obtained previously [5]. Here we compare them directly with the effects of Stöber-synthesized silica NPs SiO_2_-50 nm and additionally compare them to an ALI exposure method. The results after ALI exposure showed that both silica NPs qualitatively induced the same toxic and pro-inflammatory effects as after submerged exposure, however, quantitatively much less pronounced. During ALI exposure the particle dose was linearly applied over a time period of 5 h (Aerosil200) and 7 h (SiO_2_-50 nm) and thereafter remained constant (Figure S2, Supporting Information File 1). For submerged conditions, particles continuously settle down onto the cells in dependence of the agglomeration state and the viscosity of the medium [31–32]. Therefore, the constant mechanical stress imposed by particle deposition might explain the increased sensitivity of cells under submerged culture conditions. Indeed, the importance of shear forces to exacerbate NP-induced toxicity has been described previously [8,33]. Another explanation could also be the secretion of surfactant by A549 cells under ALI conditions [34] which may have a protective effect due to binding of surfactant proteins to the particles [35].

Furthermore, Aerosil200 NPs induced similar but more intense cellular responses compared to SiO_2_-50 nm NPs. A possible reason could be the higher specific surface area of the Aerosil200 NPs with 200 m^2^·g^−1^ compared to 60 m^2^·g^−1^ of the SiO_2_-50 nm NPs. The dependence of biological effects on particle size and specific surface area is well known and has already been shown for many types of particles including amorphous silica particles [36].

## Conclusion

In this study two silica NPs produced by different synthesis methods were intensively characterized with regard to their properties in aerosols, deposition from the aerosol onto cells and biological endpoints under ALI and submerged conditions. We could confirm our previous finding with SiO_2_ NP suspensions from another manufacturer (Ludox AS40) that the generation of an aerosol that contains monomeric silica particles is possible when using an electrospray generator [18]. Furthermore, a VITROCELL^®^ exposure module was modified and equipped with electrodes to allow electrostatic deposition enhancement by more than a factor of about 20. However, although the deposited particle number for monomeric silica particles of 53 nm diameter was comparable to those of agglomerated silica particles the mass and surface doses were too low to induce significant biological effects in lung cells. Therefore, an atomizer was used to generate aerosols with larger agglomerates allowing application of mass and surface doses that were up to three orders of magnitude higher.

Both types of silica NPs induced cytotoxicity and inflammatory responses under conventional submerged conditions. At the same nominal applied dose Aerosil200 NPs were more toxic than 50 nm silica NPs produced by the Stöber method. However, when considering the specific surface area both NPs show similar potencies. This supports previous findings that presumably the interaction of cells with the silica surface triggers adverse effects. Surprisingly, at the ALI the apparent toxicity of both NPs was drastically reduced although the deposited mass was higher by a factor of three to seven. The underlying reasons for the different sensitivity remain unknown. Future studies need to address the relevance of changes in the dose rate as a critical parameter for cellular toxicity. Moreover, more detailed analysis of particle uptake (e.g., by confocal microscopy or TEM) under ALI and submerged conditions will be helpful to unravel the different responses. This study is one of only a handful of similar attempts to directly compare biological effects of NPs under ALI and submerged conditions. In a triple-culture comprised of human lung epithelial cells, macrophages and dendritic cells were exposed to similar doses of ZnO particles under submerged and ALI conditions. Interestingly, the response (LDH release, HO-1 induction) occurred more rapidly at the ALI but to a similar extend [37]. Similarly, exposure of A549 cells to ZnO particles at the ALI provoked a stronger increase in pro-inflammatory gene expression and the concentrations of particles to induce the lowest observed effect levels were reduced [38]. Moreover, gold nanoparticles were deposited with the previous system but, in contrast to some other submerged studies, did not induce adverse effects [39]. Also a mono-culture of bronchial 16HBE14o cells induced IL-8 already at much lower diesel exhaust particle concentrations deposited at the ALI in comparison to submerged exposure [40]. ALI and submerged exposure have also recently been compared for their response to a chemical inducer of oxidative stress. In line with our findings, a tetra-culture of A549, THP-1, mast and endothelial cells reacted more sensitive under classical submerged conditions with respect to release of IL-8 and production of ROS [10]. Therefore, more NPs with different chemistries and sizes as well as different cell culture models need to be assessed in order to either confirm a reduced toxicity of NPs at the ALI in general or rather to identify a NP-specific behaviour that depends on the exposure method. Furthermore, it is possible that at the ALI gene expression will change compared to submerged cultures, a topic which warrants further investigations. Indeed, human bronchial epithelial cells are more resistant to stress imposed by ambient air pollution particles and decrease expression of the pro-inflammatory marker IL-8 and the anti-oxidant gene heme oxygenase 1 (HOX1) when cultivated at the ALI prior to exposure [41]. However, first studies in A549 cells for those selected genes do not indicate major changes in expression within a few hours of cultivation at the ALI [38].

Recently, the concept of “dose rate” as a critical driver of toxicity was again promoted [42]. In animal experiments, instillation of high doses of titania NPs initiates inflammation whereas deposition of the same mass by inhalation is almost without effect. The difference between the two approaches is a drastically increased dose rate (micrograms per minute) when using instillation versus inhalation. However, the interpretation of such in vivo experiments is complicated, because not only the dose rates differ, but also the distribution of the NPs within the lung and the clearance are different. As in our in vitro studies these two confounding factors are eliminated, the dose rate still remains an important determinant to possibly explain the differences in toxicity when exposing cells at the ALI and under conventional submerged conditions. However, in our study the dose rate is considerably lower for the submerged versus ALI deposition and therefore does not correlate with increased toxicity. This raises of course the question which dose rates are most realistic and how comparable different studies are. Presumably, the use of other ALI systems delivering the dose almost instantly (bolus deposition), and thus avoiding the necessity to incorporate sophisticated humidification systems as used in our ALIDA, will also provide different outcomes because at the even higher dose rate the relative response to NPs might change. Certainly, a systematic comparison of different air–liquid-interface methods including classical submerged toxicological assays is needed to assess the relevance of dose and dose rate and to finally relate results obtained from such in vitro methods to data obtained from exposure studies with animals or even humans.

## Experimental

### Materials

Aerosil^®^200 powder was kindly provided by Evonik (Essen, Germany). The SiO_2_-50 nm NPs without or with labelling with fluorescein isothiocyanate (FITC) supplied as a monodisperse solution of 25 mg/mL in water were from Postnova Analytics (Z-PS-SIL-GFP-0.07, Landsberg am Lech, Germany). Dulbecco’s Modified Eagle Medium (DMEM), Roswell Park Memorial Institute medium (RPMI-1640), Hank’s Balanced Salt Solution (HBSS), Dulbecco’s Phosphate Buffered Saline without Ca^2+^ and Mg^2+^ (DPBS^-/-^), penicillin, streptomycin, and trypsin were from Life Technologies (Frankfurt am Main, Germany). Fetal calf serum (FCS) was from PAA (Cölbe, Germany). The cytotoxicity detection kit for determining release of LDH was from Roche (Mannheim, Germany). Enzyme-linked immuno assays (ELISA) for the detection of human IL-6 and IL-8 were from BD Biosciences (OptEIA kits, Heidelberg, Germany). 4-(2-Hydroxyethyl)piperazine-1-ethanesulfonic acid (HEPES) and the chemicals for sodium dodecylsulfate polyacrylamide gel electrophoresis (SDS-PAGE) were from Carl Roth (Karlsruhe, Germany). For the Western blots Immobilon-P PVDF membranes (Millipore, Eschborn, Germany), primary antibodies against COX-2 (Biozol, Eching, Germany), phospho-p38 (Thr 180/ Tyr 182, Cell Signalling, Frankfurt am Main, Germany), PCNA (PC-10), Lamin B (M-20) and p38 (C-20) (both from Santa Cruz), HRP-conjugated secondary antibodies (DAKO, Hamburg) and the enhanced chemiluminescence (ECL) detection system from GE Healthcare (Freiburg, Germany) were used. Transwell^®^ inserts containing polyester membranes of 24 mm diameter and with pores of 0.4 µm (Cat.No. 3450) were from Corning Life Sciences (Amsterdam, The Netherlands). 75-mesh formvar-coated copper grids for transmission electron microscopy (TEM) were from Plano GmbH (Wetzlar, Germany).

#### Aerosol generation and characterisation

For cell exposure to nanoparticle monomers the monodisperse SiO_2_-50 nm NP suspensions were dispersed into synthetic air (Air Liquide, 20% O_2_ in N_2_, less than 3 ppm impurities) by using an electrospray aerosol generator (TSI 3480, Shoreview, United States) as described by Comouth et al. [18]. To ensure agglomerate fractions of less than 7% the suspension was diluted to 7 mg·mL^−1^ with Nanopure water (type 1 ultrapure water, Barnstead, Germany). In addition to the electrospray an Atomizer (Topas, ATM 220, Dresden, Germany) was used for aerosol generation. The particle number concentrations and size distributions of the aerosol were continuously measured by using a scanning mobility particle sizer (SMPS, TSI, Aachen, DMA 3081 and CPC 3025).

#### Exposure of cells to aerosol

The cells were exposed to the aerosol with the ALIDA exposure system described in detail by Comouth et al. [18]. For cell exposure the aerosol was humidified to 80–90% relative humidity at 37.5 °C. The humidified aerosol was then carried with identical flows of 100 mL·min^−1^ (sccm) through six tubes of equal dimensions to the inlets of six exposure chambers (VITROCELL Systems GmbH, Waldkirch). Two stainless steel modules were equipped with three Transwell^®^ inserts of 24 mm membrane diameter. To increase the deposition efficiency an almost homogeneous electrical field with an axial strength of 500 V·mm^−1^ was applied.

#### Determination of the dose

In order to determine the deposited fraction of the applied aerosol during ALI exposure TEM grids (Plano, SF162-6) were placed on the Transwell membranes at three different radial positions and exposed to the aerosol for different times. Subsequently, 50 micrographs of the deposited particles were taken for each experiment by using a transmission electron microscope (Zeiss 109T, Oberkochen). The deposited particles were detected and analysed regarding their number and size with a custom-made software.

For the calculation of mass doses spherical primary particles with a density of 2 g·cm^−3^ were assumed. This value is close to the 1.8–2.2 g·cm^−3^ stated by the manufacturer (Postnova Analytics) and to literature data for amorphous silica particles of 2.2 g·cm^−3^ [43]. For agglomerates remaining from droplets containing more than one particle an effective density cannot easily be defined. For hard spheres Manoharan et al. [27] suggested that the particles are configured in spherical packings with structures that minimize the second moment of the mass distribution. Hence we treated large agglomerates as compact spherical clusters with volume fractal dimensions close to 3. Furthermore we assumed a volume filling factor of 1.56 assuming a polytetrahedral structure. In order to justify this simplification the deposited mass dose of the FITC-labeled SiO_2_-50 nm particles was estimated additionally from their fluorescent intensity. Therefore, the exposed Transwell inserts were filled with 0.8 mL distilled water to suspend the deposited particles. The fluorescence of the solution was measured at (485 ± 10) nm excitation and (530 ± 12) nm emission wavelengths by using a Bio-Tec FL600 spectrometer and the software package KC4 (MWG-Biotech AG, Ebersberg, Germany). At the same time Transwell inserts filled with 0.8 mL of suspensions containing FITC-labeled SiO_2_-50 nm NPs at 0.25 mg·mL^−1^, 0.5 mg·mL^−1^ and 1 mg·mL^−1^ were used for calibration. Since Aerosil200 particles are not fluorescent this procedure could not be applied to verify the corresponding TEM results. In order to give at least a lower limit of the mass dose the effective density of 80 to 800 nm sized agglomerates was measured by using an Aerosol Particle Mass (APM) analyser (Kanomax, APM 3601) [44] in combination with a differential mobility analyser (DMA) connected upstream. The total amount of deposited particles was estimated finally by extrapolating the amount of deposited particles as a function of the applied particle mass.

#### Cell culture

The human alveolar epithelial cell line A549 obtained from American Type Culture Collection (ATCC, Rockville, MD) was maintained in DMEM supplemented with 10% (*v*/*v*) FCS, 2 mM L-glutamine, 100 U·mL^−1^ penicillin, and 100 mg·mL^−1^ streptomycin at 5% CO_2_ at 37 °C. The cells were passaged every three to four days. Two days before ALI exposure experiments 4 × 10^5^ cells in 1 mL of RPMI-1640 medium with 10% FCS were seeded per Transwell insert with a surface area of 4.7 cm^2^. This corresponds to a cell density of 8.5 × 10^4^ cells·cm^−2^. At the day of exposure cells formed a confluent monolayer as assessed by microscopy.

#### Submerged treatment of cells

The Aerosil200 particles were suspended in cell culture medium without 10% FCS at 10 mg·mL^−1^ and probe-sonified (Branson Sonifier, 250, Schwäbisch Gmünd, Germany) for 50 s (50 duty cycles, output 5) right before preparing dilutions and adding to cells. The SiO_2_-50 nm particles were diluted in medium and vortexed.

#### Exposure of cells under ALI conditions

Shortly before the experiment the medium above the cells and under the membrane was removed and both sides were washed with HBSS. 1.5 mL RPMI1640 medium without sodium bicarbonate, prepared from RPMI1640 powder, containing 10 mM HEPES, 100 U·mL^−1^ penicillin, 100 mg·mL^−1^ streptomycin and without FCS was pipetted into the basal compartment and the apical compartment was covered by a thin layer of 100 µL HBSS (ca. 214 µm initial mean layer height) in order to avoid drying out of the cells. RPMI instead of DMEM was used for growing cells at the ALI as it allowed a better maintenance of a stable pH which is required to keep cells viable. The cells were transported to the ALIDA system in a pre-warmed and thermally insulated box and exposed. Two inserts with cells were exposed to clean air which was generated by passing the humidified and conditioned aerosol through HEPA air filters (HepaVent™ Y271, Whatman GmbH, Dassel). Three other inserts were exposed to the unfiltered aerosol and one equipped with TEM grids for dose determination. Each exposure was done with and without applying an electrostatic field enhancing the deposition efficiency.

#### Determination of biological effects

After the ALI exposure the cells were either directly lysed or further incubated under submerged conditions in serum-free RPMI medium without HEPES at 37 °C and 95% humidity and analysed after 24 h. Post-incubation was performed submerged in order to allow optimal release of cytokines into the apical compartment. For comparison, cells grown in Transwell inserts were simultaneously treated under submerged conditions in serum-free RPMI medium for the same time periods. The cell lysates were analysed for COX-2 and phosphorylated p38 by Western blot. The media of the post-incubated cells were analysed for release of LDH by using a test kit from Roche according to the manufacturer’s instructions. Release of IL-6 and IL-8 was analysed by enzyme-linked immune assay (ELISA) kits from Becton Dickinson according to the manufacturer’s instructions.

#### Western blots

Whole cells were lysed with 2× Lämmli SDS Buffer (160 mM Tris·HCl pH 6.8, 4% SDS, 20% glycerol, 4% ß-mercaptoethanol). The cell lysates were boiled at 95 °C for 5 min, probe sonified for 15 s and then stored at −20 °C. Equal amounts of the lysates were loaded onto 10% SDS-polyacrylamide gels and after electrophoresis, the proteins were transferred onto Immobilon-P PVDF membranes. The membranes were blocked with 5% (*w*/*v*) non-fat dry milk in 1% Tween20 in Tris-buffered saline (TBS) for 1 h. For the detection of phosphorylated p38 the membranes were blocked in 3% (*w*/*v*) BSA. The blots were then incubated with primary antibodies and subsequently with HRP-conjugated secondary antibodies which were finally detected with the ECL system.

## Supporting Information

Supporting Information contains 1) data obtained by dynamic light scattering of the particles suspensions 2) data on the deposited mass dose for Aerosil200 particles after ALI exposure and 3) deposition kinetics of the mass doses for Aerosil200 and SiO_2_-50 nm particles during ALI and submerged exposure.

File 1 Additional experimental data.
