# Supplementary material for: Silica nanoparticles are less toxic to human lung cells when deposited at the air–liquid interface compared to conventional submerged exposure
Source: Beilstein J Nanotechnol. 2014 Sep 19;5:1590–602. doi: 10.3762/bjnano.5.171 (PMC4168966; doi:10.3762/bjnano.5.171)
Supplement: File 1 — Additional experimental data. [file Beilstein_J_Nanotechnol-05-1590-s001.pdf]

Supporting Information

for

Silica nanoparticles are less toxic to human lung cells when deposited at the air–liquid interface compared to conventional submerged exposure

Alicja Panas[1‡], Andreas Comouth[2‡], Harald Saathoff[2], Thomas Leisner[2], Marco Al-Rawi[1], Michael Simon[3], Gunnar Seemann[3], Olaf Dössel[3], Sonja Mülhopt[4], Hanns-Rudolf Paur[4], Susanne Fritsch-Decker[1], Carsten Weiss*[1§] and Silvia Diabaté*[1,¶]

Address: [1]Institute of Toxicology and Genetics, Karlsruhe Institute of Technology, Campus North, Hermann-von-Helmholtz-Platz 1, 76344 Eggenstein-Leopoldshafen, Germany; [2]Institute for Meteorology and Climate Research, Karlsruhe Institute of Technology, Campus North, Hermann-von-Helmholtz-Platz 1, 76344 Eggenstein-Leopoldshafen, Germany; [3]Institute of Biomedical Engineering, Karlsruhe Institute of Technology, Campus South, Kaiserstraße 12, 76128 Karlsruhe, Germany and [4]Institute for Technical Chemistry, Karlsruhe Institute of Technology, Campus North, Hermann-von-Helmholtz-Platz 1, 76344 Eggenstein-Leopoldshafen, Germany

Email: Silvia Diabaté* - silvia.diabate@kit.edu; Carsten Weiss* - carsten.weiss@kit.edu

*Corresponding author

[‡]These authors contributed equally to the paper.

[§]Tel: +49 (0)721-608 24906

[¶]Tel: +49 (0)721-608 22692

Additional experimental data

Supplementary Methods

Dynamic light scattering

Particles were suspended in deionized water at 10 mg/ml, shortly vortexed and probe sonified with 30 strokes, 50% cycle duty, output control: 8 (Branson Sonifier 250, Schwäbisch Gmünd, Germany). This suspension was further diluted in deionized H_2O to 1 mg/ml. For DLS analysis, SiO_2-NPs were further diluted to 50 µg/ml in deionized H_2O or DMEM without serum. The samples were either analysed directly (0h) or after incubation at 37°C and 5% CO_2 for 24 h immediately after vortexing using the Zetasizer Nano ZS (Malvern Instruments Ldt., Herrenberg, Germany) at 25°C.

Supplementary Table

Table S1: Characterisation of SiO_2-NP suspensions. The hydrodynamic diameters (d_H) were analysed in water and in DMEM without serum at a NP concentration of 50 µg/ml directly after dispersion (0h) and after incubation at 37°C and 5% CO_2 for 24h and vortexing. Data are means ± s.e.m. of three measurements.

Particle	Medium	Time (h)	d_H [nm]
Aerosil200	H_2O	0<br>24	236 ± 9<br>233 ± 19
Aerosil200	DMEM	0<br>24	220 ± 6<br>225 ± 7
SiO_2-50 nm	H_2O	0<br>24	74 ± 22<br>72 ± 20
SiO_2-50 nm	DMEM	0<br>24	72 ± 19<br>65 ± 30<br>2178 ± 1373

Supplementary Figures

Determination of the deposited mass dose for Aerosil200 particles after ALI exposure

Determination of the mass dose for Aerosil200 particles is complex since they are not only composed of a broad size distribution of agglomerates but these agglomerates are also composed of monomers with a broad size distribution. Furthermore, the agglomerates are

not as compact as those composed of the SiO_2-50nm particles. If we analyse the TEM micrographs loaded with Aerosol200 particles in the same way as for the compact SiO_2-50nm agglomerates the corresponding deposited mean mass dose is about 78 µg cm^{-2}. However, in this case this value is an upper limit for the actual mass dose since the Aerosil200 agglomerates show a more complex cluster structure compared to SiO_2-50nm agglomerates that cannot be described by a packing of hard spheres. In fact the effective densities of Aerosil200 agglomerates decrease with increasing agglomerate size. We used an aerosol particle mass analyser (APM) in combination with a differential mobility analyser (DMA) to determine the effective density of the Aerosol200 agglomerates as a function of their mobility equivalent size. As shown in Figure S1 this dependence can be well described by a bi-exponential function.

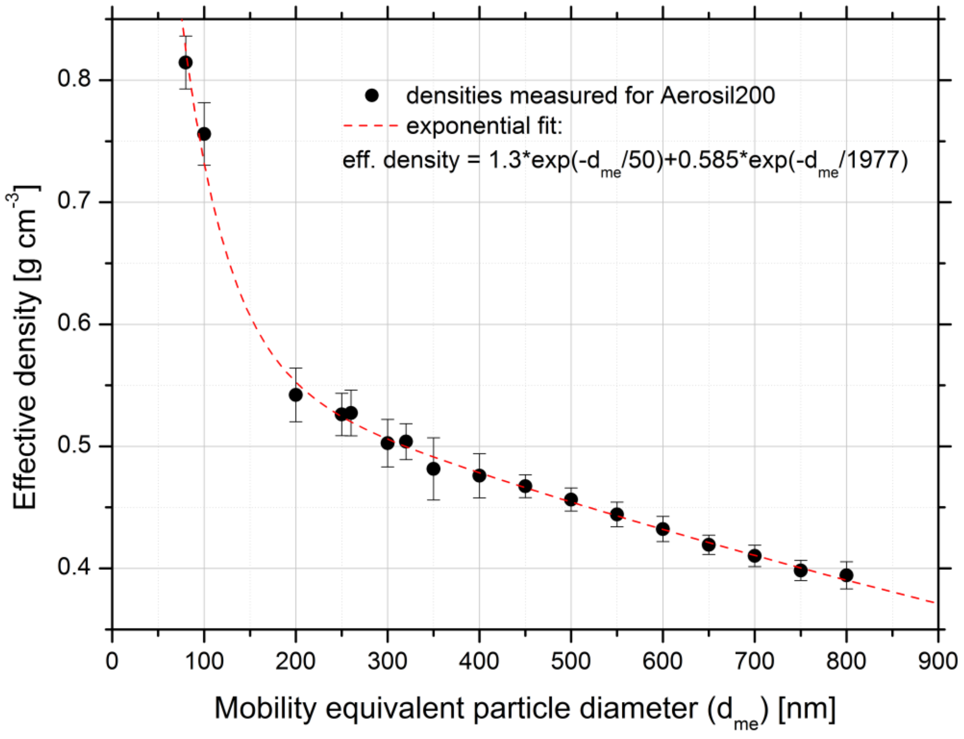

Figure S1: Effective densities measured for Aerosol200 dispersed with an atomizer by combination of a differential mobility analyser (DMA) and an aerosol particle mass analyser (APM). The observation is well represented by a bi-exponential function.

Since 90% of the detected particles on TEM micrographs were smaller than 800 nm in projected area equivalent diameter and the decrease in effective density becomes small for large particles we estimate a lower limit for the real mass dose to about 26 µg cm^{-2} by using an average density of 0.4 g cm^{-3}. A more accurate estimation however requires a better understanding of the correlation between the projection equivalent diameter, mobility

equivalent diameter and effective density and has to be part of future work. Nevertheless, the real mass dose has to be between the limits determined above and can be estimated to (52 ± 26) µg cm^{-2}.

Deposition kinetics of the mass doses for Aerosil200 and SiO2-50 nm particles during ALI and submerged exposure

The ***In** vitro* **S**edimentation, **D**iffusion and **D**osimetry model (ISDD) developed by Hinderliter at al. (1) was used to calculate the particle mass which is delivered to the cell surface under submerged exposure in serum-free cell culture medium containing 50 µg ml^{-1} of particles over 24 h. For the calculation we used the measured hydrodynamic diameters in DMEM after 24 h which were 225 ± 7 nm for Aerosil200 and 65 ± 30 nm and 2178 ± 1373 nm for SiO_2-50nm particles (see Table S1). Based on DLS measurements of the SiO_2-50nm particle suspension, we estimate that 90% of the mass is present in the fraction of the small particles and 10% in the fraction of the large agglomerates.

During ALI exposure the deposited dose increases linearly until 5 h (Aerosil200) and 7 h (SiO2-50 nm) and remains constant during the post-incubation period until 24 h. (Figure S2-A). The dose values were taken from Table 1 and the way how the dose was determined is described in the text.

Under submerged conditions the cellular doses of Aerosil200 and SiO2-50 nm increase continuously over 24 h as shown in Figure S2-A and in a magnification of the lower part of the diagram in Figure S2-B. For both particles the calculated cellular dose reaches 7 µg cm^{-2} after 24 h.

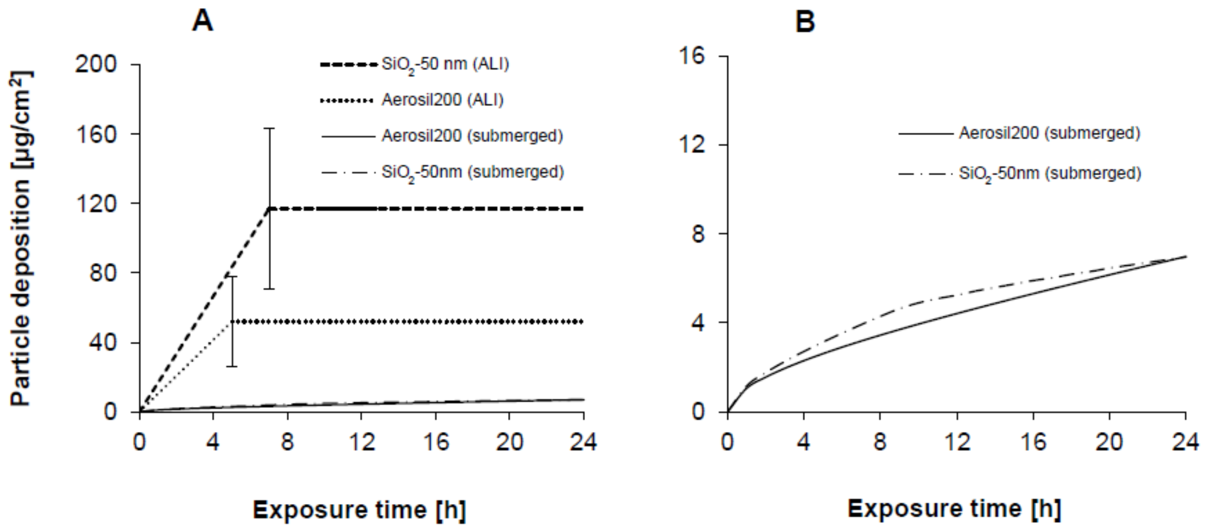

Figure S2: Kinetics of the mass doses for Aerosil200 and SiO_2-50 nm particles after ALI and submerged exposure over 24 h (A). The doses for ALI exposure were determined experimentally (see text) and the doses for submerged exposure were calculated by the ISDD model. (B) shows a magnification of the lower part of diagram (A).

References

1. Hinderliter, P. M.; Minard, K. R.; Orr, G.; Chrisler, W. B.; Thrall, B. D.; Pounds, J. G.; Teeguarden, J. G. *Part Fibre Toxicol.* **2010,** *7,* 36.
